# First Detection of *Human Coronavirus HKU1* in Greece, in an Immunocompromised Patient With Severe Lower Respiratory Tract Infection

**DOI:** 10.15388/Amed.2021.28.1.21

**Published:** 2021-05-14

**Authors:** Vasiliki Epameinondas Georgakopoulou, Georgios Petsinis, Konstantinos Mantzouranis, Christos Damaskos, Despoina Melemeni, Aikaterini Gkoufa, Serafeim Chlapoutakis, Nikolaos Garmpis, Pagona Sklapani, Nikolaos Trakas, Xanthi Tsiafaki

**Affiliations:** Pulmonology Department, Laiko General Hospital, Athens, Greece; 1^st^ Pulmonology Department Sismanogleio Hospital, Athens, Greece; 1^st^ Pulmonology Department Sismanogleio Hospital, Athens, Greece; Second Department of Propedeutic Surgery, Laiko General Hospital, Medical School, National and Kapodistrian University of Athens, Athens; Greece N.S. Christeas Laboratory of Experimental Surgery and Surgical Research, Medical School, National and Kapodistrian University of Athens, Athens, Greece; ^2 ^1^st^ Pulmonology Department Sismanogleio Hospital, Athens, Greece; First Department of Internal Medicine, Laiko General Hospital, Medical School, National and Kapodistrian University of Athens, Athens, Greece; Department of Thoracic Surgery, Agios Savvas Hospital, Athens, Greece; Second Department of Propedeutic Surgery, Laiko General Hospital, Medical School, National and Kapodistrian University of Athens, Athens Greece; N.S. Christeas Laboratory of Experimental Surgery and Surgical Research, Medical School, National and Kapodistrian University of Athens, Athens, Greece; Department of Cytology, Mitera Hospital, Athens, Greece; Department of Biochemistry, Sismanogleio Hospital, Athens, Greece; 1^st^ Pulmonology Department Sismanogleio Hospital, Athens, Greece

**Keywords:** HCoV-HKU1, Human Coronaviruses, Pneumonia, Immunosuppression

## Abstract

*Human coronavirus HKU1* (*HCoV-HKU1*) is a RNA virus which gets in the human cells by binding to the receptor of N-acetyl-9-O-acetylneuraminic acid.* Human Coronaviruses (HCoVs*), including *HCoV-HKU1*, are globally found. *HCoV-HKU1 *is responsible for upper and lower respiratory tract infections, usually with mild symptoms. In severe cases, *HCoV-HKU1 *can cause life-threatening respiratory illness especially in vulnerable hosts such as elderly, children and immunocompromised patients. In Greece, *Respiratory Syncytial Virus (RSV)* and *influenza* are the most common viruses causing respiratory tract infections. Traditionally, *HCoVs *are responsible for less than 3% of respiratory infections in Greek population. HCoVs 229E and OC43 have been shown to circulate in Greece. We report the first case of lung infection in an immunocompromised woman due to *HCoV-HKU1, *that has never been before detected in Greece. *HCoV-HKU1* is related to severe disease even in healthy individuals and must be considered in the differential diagnosis of severe respiratory infections.

## Abstract (lt)

**Pirmasis žmogaus koronaviruso *HKU1* atvejis Graikijoje, nustatytas pacientui, turinčiam imuninės sistemos sutrikimą ir sergančiam sunkia apatinių kvėpavimo takų infekcija:**

**Santrauka. **Žmogaus koronavirusas* HKU1* (*HCoV-HKU1*) yra *RNR* virusas, jis patenka į žmogaus organizmo ląsteles, jungdamasis prie N-9-O-acetilneuramino rūgšties receptoriaus.

Žmogaus koronavirusai* (hCoVs*), įskaitant *HCoV-HKU1*, yra aptinkami visame pasaulyje. *HCoV-HKU1 *sukelia viršutinių ir apatinių kvėpavimo takų infekcijas, tačiau paprastai simptomai yra nežymūs. Sudėtingu atveju *HCoV-HKU1*gali sukelti gyvybei pavojingą kvėpavimo takų ligą, ypač asmenims, priklausantiems pažeidžiamoms asmenų grupėms, pavyzdžiui, vyresnio amžiaus žmonėms, vaikams ir pacientams, kurių imuninė sistema yra sutrikusi. Graikijoje respiracinis sincitinis virusas* (RSV)* ir gripo virusas yra dažniausi virusai, sukeliantys kvėpavimo takų infekcijas. Tradiciškai *hCoVs *sukelia mažiau negu 3 % kvėpavimo takų infekcijų Graikijos populiacijoje. Paaiškėjo, kad virusai *HCoVs 229E* ir *OC43* vyrauja Graikijoje. Straipsnyje pristatome pirmąjį plaučių infekcijos, kurią sukėlė *HCoV-HKU1 *moteriai, turinčiai sutrikusį imunitetą, atvejį, tokio niekada anksčiau nebuvo nustatyta Graikijoje. *HCoV-HKU1 sukelia sunkią ligą net ir sveikiems asmenims ir į tai būtina atsižvelgti diferencinėje sunkių kvėpavimo takų infekcijų diagnostikoje.*

**Reikšminiai žodžiai: ***HCoV-HKU1*, žmogaus koronavirusai, pneumonija, imunosupresija

## Introduction

*Human coronavirus HKU1* (*HCoV-HKU1*) is a coronavirus species in humans. It is an enveloped, RNA virus which gets in the human cells by binding to the receptor of N-acetyl-9-O-acetylneuraminic acid [1]. It has the Hemagglutinine estarase (HE) gene, which differentiates it as a member of the genus *Betacoronavirus* and subgenus *Embecovirus* [2]. 

*HCoV-HKU1 *was first described in a 71-year-old man who had returned to Hong Kong from Shenzhen, China in 2004 and developed bilateral pneumonia and acute respiratory distress syndrome [3].* Human Coronaviruses (HCoVs*), including *HCoV-HKU1*, are globally found in the humans and are responsible for approximately one-third of human common cold infections. In severe cases, they can cause life-threatening pneumonia and bronchiolitis especially in vulnerable hosts such as elderly, children and immunocompromised patients. In addition to respiratory diseases, they also cause gastrointestinal and neurological illnesses [1,4]. 

In Greece, according to large epidemiological studies, *Respiratory Syncytial Virus (RSV)* and *influenza* are the most common viruses responsible for respiratory tract infections. Traditionally, *HCoVs *are responsible for less than 3% of respiratory infections in Greek population [5,6]. We report the first case of lung infection in an immunocompromised woman due to *HCoV-HKU1, *that has never been before detected in Greece.

## Case Report

A 67-year-old woman, with a history of arterial hypertension, diabetes mellitus type II, thyroidectomy, osteoporosis, hyperlipidemia and rheumatoid arthritis being treated with methotrexate and corticosteroids, presented to our Pulmonology Department in August 2019 with progressive dyspnea at rest and fatigue over the last two days. 

**Figure 1.**
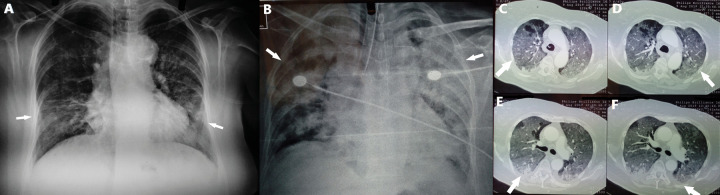
A: Chest X-ray shows patchy diffuse infiltrates in both lungs mostly in the left lower lobe on admission. B: *Chest X-ray shows diffuse lung infiltrates after two days of hospitalization.* *C, D: *Chest Computed Tomography reveals diffuse ground glass opacities. E, F: Chest Computed Tomography reveals pleural effusion in both lungs.

Clinical examination revealed an afebrile patient with crackles on auscultation at all lung fields. Blood pressure was 100/80 mmHg, heart rate was 120 beats per minute, oxygen saturation was 79% on room air and body temperature 36.5 °C on admission. Electrocardiography showed sinus tachycardia. Arterial blood gas analysis revealed pO_2 _40mmHg, pCO_2 _35mmHg, pH 7.48 and HCO_3_¯ 29.2 mmol/L on room air. Chest X-ray showed patchy diffuse infiltrates in both lungs mostly in the left lower lobe (Figure 1A).

Complete blood count was normal. The blood biochemistry parameters and thyroid-stimulating hormone (TSH) were normal, with the exception of an elevated serum lactate dehydrogenase (LDH) 311 U/L (normal <225 U/L), serum glycose 263 mg/dl (normal 60-100 mg/dL) and C-reactive protein (CRP) 122.3 mg/L (normal <6 mg/L). Urinalysis was normal. 

The reversed transcription polymerase chain reaction (RΤ-PCR) test for Influenza A and B in nasopharyngeal and oropharyngeal samples were performed and were negative. Urinary antigen tests for *Legionella pneumophila *and *Streptococcus pneumoniae*, serological testing for *Mycoplasma pneumonia* and blood cultures were negative. 

The patient received oxygen therapy with Venturi mask delivering 60% oxygen and intravenous antibiotic therapy with ceftriaxone, azithromycin and trimethoprim-sulfomethoxazole empirically. After the first two days of hospitalization the patient presented with respiratory deterioration and diffuse lung infiltrates (Figure 1B), was intubated and admitted to intensive care unit.

Computed tomography (CT) of the chest was performed. Chest CT revealed diffuse ground glass opacities in all lung fields (Figure 1C, 1D) and bilateral pleural effusion (Figure 1E, 1F). The patient underwent echocardiography with normal ejection fraction and valve function. Bronchoscopy was performed and bronchial washings and bronchoalveolar lavage (BAL) were obtained from lingula and right middle lobe. Cytological and microbiological examination of bronchial washings and BAL was negative. Direct immunofluorescence and PCR for *Pneumocystis jirovecii *in BAL were negative and administration of trimethoprim-sulfomethoxazole was discontinued. Respiratory virus panel test in BA for detection of human respiratory viruses by use of multiplex RT-PCR was performed and revealed *HCoV-HKU1. *

The patient after five days of intubation presented with improvement, was extubated and received oxygen therapy with Venturi mask. The patient had gradually completely recovered without any specific therapy. She denied recent travel or contact with a person that had recently returned from a travel abroad. 

## Discussion

*HCoV-HKU1* is related to both upper and lower respiratory tract infections*. *The most common method for diagnosing *HCoV-HKU1* infection is RT-PCR or real-time RT-PCR using RNA extracted from respiratory tract samples. *HCoV-HKU1* infections have the highest incidence in winter, however they occur also in spring and summer [7]. *HCoV-HKU1-*associated pneumonia is a monophasic disease, and most patients have mild symptoms that were localized to the respiratory tract. Most patients with *HCoV-HKU1-*associated pneumonia are >65 years old. There is currently no treatment recommended for* HCoV-HKU1-*associated pneumonia except for supportive care as needed [8].

*HCoV-HKU1 *has been associated with acute respiratory illness in patients with underlying disease such as immunosuppression. Fatal pneumonia due to *HCoV-HKU1 *has been described in patients with diabetes mellitus, malignancy and cardiovascular disease [8]. *HCoV-HKU1 *has been detected as causative agent of severe respiratory infection in older adults with Chronic Obstructive Pulmonary Disease (COPD) [9]. Lower respiratory tract infections due to *HCoV-HKU1* in patients with hematopoietic stem cell transplantation or hematological malignancies are associated with high rates of oxygen use and mortality [10]. In addition, *HCoV-HKU1 *has been reported to cause diffuse pneumonitis in a 65-year-old man diagnosed with stage IV melanoma who developed pulmonary and brain metastases and was treated with combined nivolumab and ipilimumab immunotherapy [11]. 

*HCoV-HKU1 *is considered to have global distribution with a median incidence of 0.9 (0–4.4) % [7, 12]. It has been described to be responsible for 2.1% of respiratory infections due to *HCoVs* in Kenya during 2009-2012 [13], 16.6% of respiratory infections due to *HCoVs* in Yamagata, Japan during 2010-2019 [14], 0.13% of respiratory infections due to *HCoVs *in Hong Kong during 2008-2014 [15], 7.82% of respiratory infections due to *HCoVs* in Guangzhou, China during 2010–2015 [16], 1.6% of all respiratory infections in Cleveland, Ohio in 2016 [17], 1.1% of all respiratory infections in Kuala Lumpur, Malaysia [18]**, **0.6% of respiratory infections due to *HCoVs *in the United States during 2014–2017 [19] and 0.32% of respiratory infections due to *HCoVs *in Thailand during 2012–2013 [20]. 

*HCoV-HKU1, HCoV 229E, NL63 *and* OC43* are known as nonsevere acute respiratory syndrome (SARS)-like *CoVs* while the other three HCoVs, SARS-CoV-1, MERS-CoV and SARS-CoV-2 are thought to be highly pathogenic, causing lethal human disease [12]. However, *HCoV-HKU1 *has been associated with severe and fatal respiratory infection in patients without underlying diseases, between 2013 and 2017, in Brazil [21] and with severe acute respiratory illness among patients hospitalized in South Africa during 2012-2013 [22].

In addition, *HCoV-HKU1 *has been detected in patients with respiratory infections in European countries. *HCoV-HKU1 *was detected in nasal samples and stool samples from patients hospitalized for respiratory infection in February and March 2005 at the University Hospital of Caen, France [23]. Moreover, *HCoV-HKU1 *was the cause of acute respiratory tract infections in patients hospitalized in Pavia, Italy during the period January-May 2006 [24]. 

In Greece, data about epidemiological and clinical aspects of *HCoVs *are limited.

HCoVs 229E and OC43 have been shown to circulate in Greek population [5]. To our knowledge, *HCoV-HKU1* has never been before detected in Greece. A further investigation about clinical and molecular epidemiology of *HCoVs *is needed in Greece. 

## Conclusions

This is the first case of isolation of *HCoV-HKU1 *in a patient with pneumonia in Greece. *HCoVs *are a common cause of respiratory infections, responsible for considerable morbidity and hospitalization of all age groups, especially in immunocompromised patients. Moreover, it is of great importance to include Coronaviruses in diagnostic panels used by official surveillance systems because along with their pandemic potential, endemic *HCoVs, *including *HCoV-HKU1,* are related to severe disease even in healthy individuals and must be considered in the differential diagnosis of severe respiratory infections. 

## Conflicts of Interest

None
